# Work-Family Conflict and Work Exit in Later Career Stage

**DOI:** 10.1093/geronb/gby146

**Published:** 2018-11-28

**Authors:** Baowen Xue, Maria Fleischmann, Jenny Head, Anne McMunn, Mai Stafford

**Affiliations:** 1 Department of Epidemiology and Public Health; 2 MRC Unit for Lifelong Health and Ageing, UCL, UK

**Keywords:** Cause-specific Cox models, Family interference with work, Gender differences, Work interference with family

## Abstract

**Objectives:**

This study investigated relationships between work–family conflict and routes of later-life work exit.

**Methods:**

We used a cohort of British civil servants (5,157 men; 2,027 women) who participated in the Whitehall II study. Work interference with family (WIF) and family interference with work (FIW) were measured up to three times over 10 years. Cause-specific Cox models were used to assess the influence of WIF/FIW on particular routes (“retirement,” “health-related exit,” “unemployment,” or “homemaker/other”) of work exit in later career stage and all routes combined.

**Results:**

WIF was not associated with any route of work exit in men or women, after adjusting for confounders. For perceived higher FIW, men were less likely to exit work through retirement, homemaker/other, or all routes combined. This was not attenuated by adding family factors or working conditions. Women with higher FIW were more likely to exit through the homemaker route. This was no longer significant after adjusting for family factors. Neither FIW nor WIF was associated with health-related exit or unemployment.

**Discussion:**

FIW makes women more likely to become a homemaker at later career stage but reduces the risk of leaving work for men, which may reinforce gender inequality in work participation.

The population in most western societies has been aging for the last few decades. Resulting pressures on social benefits systems have increased interest in explaining when and why older people leave work. Previous studies have asserted the importance of the family sphere for work and retirement, but most have investigated objective characteristics, such as marital status or the number of dependent children in the household (Stafford et al., 2018; Wahrendorf, Zaninotto, Hoven, Head, & Carr, 2017). Another way in which the family and work sphere are interdependent is expressed by work–family conflict. A conflict between the work and family sphere can be a source of stress and may influence individuals’ well-being and behavior (Geurts, Kompier, Roxburgh, & Houtman, 2003). Perceived work–family conflict has been associated with several labor market-related outcomes, such as job satisfaction (e.g. Anafart, 2011; Grandey, Cordeiro, & Crouter, 2005), intention to quit (e.g. Forma, 2009; Shaffer, Harrison, Gilley, & Luk, 2001), sickness absence (e.g. Jansen et al., 2006), or nonattendance (e.g. Boyer, Maertz, & Pearson, 2005). Even though these links are well established, few studies have so far investigated the relation of work–family conflict with retirement or other routes of later work exit. This is surprising, especially given the abundance of research showing that unfavorable work characteristics are linked to early retirement (e.g. Carr et al., 2016; Hintsa et al., 2015). In this article, we investigate the relationship between work–family conflict and routes of work exit in later career stage among men and women of the British Whitehall II occupational cohort study.

## Work–Family Conflict

Work and family may, at times, intrude on one another with one domain spilling over onto the other (Greenhaus & Beutell, 1985). Work–family conflict is defined as a form of inter-role conflict in which the role pressures from the work and family domain are mutually incompatible in some respects (Greenhaus & Beutell, 1985, p. 77). This incompatibility can be bidirectional: work responsibilities can interfere with the family sphere (WIF), for example, if an extensive workload reduces family time. Or, family responsibilities can interfere with work (FIW), for example, if caring for an older relative is incompatible with work meetings (Frone, Russell, & Cooper, 1992). WIF (work-to-family conflict) and FIW (family-to-work conflict) have been described as conceptually different constructs (Duxbury, Higgins, & Lee, 1994; Frone et al., 1992). These two constructs have, to some extent, been shown to have different antecedents. WIF was more strongly related to work-related factors than FIW, such as job stress and schedule flexibility, whereas predictors of FIW were mainly in the family domain, such as number of children and hours spent on housework (Byron, 2005).

## Work–Family Conflict and Work Exit

Previous research provides abundant examples of how family life, as well as stress perceived in the workplace, influence retirement. For example, older workers’ caring responsibility toward elderly relatives (Beehr, Glazer, Nielson, & Farmer, 2000; Dentinger & Clarkberg, 2002), having had a child late or financially dependent children in the household (Damman, Henkens, & Kalmijn, 2011) have all been shown to be related to earlier retirement. Many of these family characteristics are also predictors of FIW, suggesting that spillover of family stress into work may be an important mediator of the association between family environment and preferences to retire (Raymo & Sweeney, 2006). For example, caregiving responsibility may influence work performance, leading to an increased desire among caregivers to stop working. In terms of workplace characteristics, growth opportunities at work have been found to delay retirement (van Solinge & Henkens, 2014), whereas effort–reward imbalance and low job control relate to early retirement (Fleischmann, Carr, Stansfeld, Xue, & Head, 2017; Siegrist et al., 2007). Some of these job characteristics have been identified as antecedents of WIF, suggesting that part of the observed relationship between work characteristics and retirement may be due to the spillover of work-related stress into family life (Byron, 2005).

The focus on work–family conflict as an indicator for later-life work exit fits well with the life course perspective, which emphasizes the interdependence of life spheres in shaping outcomes across the life course (Elder, 1994). However, most research has studied work and family independently, and we could only identify five previous studies investigating the link between WIF/FIW and work exit among middle-aged or older workers (Forma, 2009; Garcia, Milkovits, & Bordia, 2014; Greenhaus, Parasuraman, & Collins, 2001; Kubicek, Korunka, Hoonakker, & Raymo, 2010; Raymo & Sweeney, 2006). Most have found that WIF and FIW were related to work exit intentions, but results for actual work exit were less consistent. On the basis of only one wave of the Wisconsin Longitudinal Study, Raymo and Sweeney (2006) found that higher levels of WIF and FIW were related to stronger preferences to retire among individuals aged 52–54. Using a cross-sectional survey from Finland, Forma (2009) found that both men and women who reported that they were neglecting home matters because of their job were more likely to consider leaving work before retirement age. Adjusting for job characteristics and family characteristics did not totally remove this association. Garcia and colleagues (2014) used a small cross-sectional Australian sample and showed that the association between FIW/WIF and preferences to continue working was mediated by lower self-efficacy. Only two prior studies have focused on actual work exit. Kubicek and colleagues (2010) used two waves from the Wisconsin Longitudinal Study and found that higher FIW decreased the probability of retiring early, whereas higher WIF was related to a higher probability of retiring early. They showed that WIF and FIW only influenced retirement timing indirectly through quality-of-life measures (i.e. marital satisfaction, job satisfaction, and health). Greenhaus and Parasuraman (2001) used a small sample of about 200 employees in public accounting. They found that neither FIW nor WIF was related to actual employment withdrawal behavior, although WIF was associated with increased withdrawal intention.

## Gender Differences in Work–Family Conflict and Work Exit

Traditionally, men were primarily involved in the work sphere and responsible for generating the family’s income, whereas women were mainly active in the family sphere, doing the childrearing and household tasks (Pleck, 1977). Over the last several decades, women have established themselves in the work sphere, but men have been much slower to take up more domestic labor (Sullivan, 2000). Even working women are often found to be primarily responsible for the family (Bartley, Blanton, & Gilliard, 2005). In this context, several related arguments exist proposing gender differences with regard to family and work life and their possible conflict. First, it is argued that the importance—or orientation, centrality, salience—of the work and family roles may differ for men and women (Noor, 2004), with family roles having a higher salience than work roles for women, and vice versa for men. A historical perspective suggests that women had higher levels of FIW, whereas men experienced higher WIF (Pleck, 1977). A meta-analysis supports this assumption, although the differences between men and women are relatively small (Byron, 2005). On the other hand, women may experience higher work–family conflict compared to men because their combined work and family demands are higher (Frone et al., 1992). Empirical support for this argument is, however, scarce. A meta-analysis suggests that women do not experience higher levels of work–family conflict than men (Shockley, Shen, DeNunzio, Arvan, & Knudsen, 2017).

In addition, men and women often have different life course attachments to the labor force. When men leave their jobs, they are exiting from a role that has typically dominated their whole adulthood. Women, however, commonly experience greater discontinuity, moving in and out of the labor force and part-time jobs, together with shifting household responsibilities (Lacey et al., 2015; Levy, Gauthier, & Widmer, 2013). Therefore, work exit may well have different meanings for men and women, and women may be more likely to exit work when there is a conflict between work and family. However, the five previous studies investigating the link between WIF/FIW and work exit have found little evidence for gender differences (Forma, 2009; Garcia et al., 2014; Greenhaus et al., 2001; Kubicek et al., 2010; Raymo & Sweeney, 2006).

## Work–Family Conflict and Routes of Work Exit

Gendered work histories might set men and women up to take different routes to exit the labor market. Given women’s less stable employment histories and occupational segregation by gender, women are less likely than men to be covered by a pension. Therefore, women may be more likely than men to exit work through routes such as domestic work rather than retirement (Wahrendorf et al., 2017), especially when they are experiencing work–family conflict. Second, work–family conflict has been associated with depression (Geurts et al., 2003) and sickness absence (Jansen et al., 2006), as a result, higher levels of work–family conflict may be linked with health-related reasons for exiting work for older people who are less able to continue work due to poor health. Sometimes, work–family conflict may be also linked with involuntary work exits. Higher levels of work–family conflict could negatively influence people’s job performance (Gilboa, Shirom, Fried, & Cooper, 2008) and increase absenteeism (Boyer et al., 2005), which may increase people’s risk of being unemployed. Therefore, work–family conflict may not only influence older people’s decision on whether to exit work, but also on the route of exit. People who exit work early or exit through routes rather than retirement may not accumulate sufficient income after exiting work, which might be especially relevant to people experiencing work–family conflict before reaching the State Pension Age. Despite the potential importance of the different routes of work exit for older people, previous studies on work–family conflict have focused on retirement only.

## The Present Study

Work–family conflict has become an increasingly salient question in the context of increasing female labor market participation; many employed adults are part of dual-earner families and families facing caring responsibility (McMunn et al., 2015). The Government’s desire to extend working lives is one of the most significant policy developments in recent times. Decisions on retirement age and retirement policy have the potential to increase health and wealth but also may widen inequalities. Work–family conflict may contribute to the context in which work exit decisions are made, including both timing and route of work exit. Understanding how work–family conflict influences work exit could help to tailor strategies to enable fuller working lives.

Most studies of work–family conflict have focused on prime working age. This study extends that literature to investigate whether work–family conflict is important across working life and continues to be salient for older workers. Prior studies either used cross-sectional data (Forma, 2009; Garcia et al., 2014; Greenhaus et al., 2001; Raymo & Sweeney, 2006) or relied on a single time-point measure of work–family conflict (Kubicek et al., 2010); in addition, most tested the intention of work exit for older people, which could be different from actual work exit. This research investigates work–family conflict, specifically WIF and FIW, in relation to actual work exit in the later career stage. Using data from a large British occupational cohort study with a more than 20 years follow-up, we aim to add to existing literature in several ways. First, we measure work–family conflict repeatedly (up to three times over 10 years) from individuals’ midlife onward and use these time-varying data to predict work exit in later career stage. Second, we differentiate various possible main routes through which older workers can leave work, namely “retirement,” “health-related work exit,” “unemployment,” and “homemaker/other exit.” We, thus, could provide insights into work exit routes that are independent of institutional regulations or pertain to different eligibility criteria. Third, we study men and women separately because they may respond to work–family conflict differently, and some routes of work exit might be more prevalent among women than men, for example, becoming a homemaker.

If the work sphere and family sphere are experienced to be incompatible, work exit could be a potential solution to work and family conflict. We hypothesize that higher work–family conflict (both FIW and WIF) is associated with increased risk of every particular type of work exit and all routes combined work exit at later career stage (Hypothesis 1). Because women were primarily involved in the family domain, especially for this relatively old birth cohort, we further hypothesize that women are more likely to exit work than men when there is a work and family conflict, especially through the homemaker route (Hypothesis 2). High levels of work–family conflict may influence people’s own health, which could in turn influence people’s work exit decisions. We hypothesize that depression and number of chronic conditions are potential mediators in the association between work–family conflict and work exit (Hypothesis 3). In addition, we hypothesize that stressful working conditions and family characteristics are sources of work–family conflict (i.e. work–family conflict mediates the relationship between stressful work and family characteristics and work exit), so relationships between work–family conflict and work exit should, therefore, be attenuated when sources of work–family conflict are controlled (Hypothesis 4).

## Method

### Sample

This study used data from a prospective occupational cohort study—the Whitehall II study. All civil servants aged 35–55 years working in the London offices of 20 Whitehall departments in 1985–1988 were invited to participate. The response rate was 73%, and a sample of 6,895 men and 3,413 women were recruited in phase 1. Follow-up surveys were conducted every 2–3 years (Marmot & Brunner, 2005); the most recent round of data collection used in this article is phase 11 (2012–2013). All participants provided written consent, and the University College London ethics committee approved this study.

Our analytic sample refers to participants who were working at phase 3 and have at least one (and up to three) valid measures of both WIF and FIW from phases 3 (1991–1994), 5 (1997–1999), and 7 (2002–2004). We use phase 3, the phase in which work–family conflict was measured first, as the baseline, and use participants’ employment information up to phase 11. From the phase 3 sample of 8,815, we first excluded 615 people who had left work before or at phase 3 (and did not go back to work later). We then excluded 244 people who reported both WIF and FIW as not applicable throughout. A total of 772 participants were excluded due to missing data (one or more items missing) in work–family conflict scales in all the phases. The analytic sample is 7,184 (5,157 men and 2,027 women) with an average age of 48.94 (standard deviation [*SD*] = 5.58) and 49.70 (*SD* = 5.84) at phase 3 and 69.51 (*SD* = 5.62) and 70.34 (*SD* = 5.85) at phase 11 for men and women, respectively.

### Measures

#### Exit from work

Respondents’ employment status was determined by self-report (“working,” “retired,” “unemployed and seeking work,” and “other”). Participants who were no longer working at a subsequent follow-up were classified as having left work (i.e. event has occurred). Participants who were still working (either in the civil service or outside) at the end of follow-up or left the study before leaving work were treated as right-censored. For people who re-entered the labor market (614 men and 182 women), the employment status of the final interview (whether working or not) was used.

#### Route of exit from work

Participants who were not working during follow-up indicated the reason for not working as “long-term sick,” “retired,” “unemployed and seeking work,” or “homemaker/other reasons”. In addition, those who were “retired” could indicate whether this was “retirement on health grounds.” We thus derive four routes of exit from paid work: “retirement” (reports being retired and not retired on health grounds), “health-related exit” (reports being long-term sick or retired on health grounds), “unemployment,” and “homemaker/other exit.” For participants who had multiple exits in the follow-up (e.g. first exit work, then re-enter the labor force, and then exit again), the route of the final exit was used.

#### Age of exit from work

For participants exiting from the civil service, we calculated participants’ exit age based on year of birth and year of exit from employment. If participants’ exact exit year was unknown (due to exit from subsequent non-civil service job or missing information), we used the midpoint between their ages in the last phase still in work and the first phase out of work. For those whose midpoint could not be calculated due to nonresponse, their current age at the first observed phase out of work was used as exit age. Again, for participants who had multiple exits in the follow-up, the age of the final exit was used.

#### WIF and FIW

Work–family conflict was measured at phases 3, 5, and 7. The extent to which one’s family interfered with work was measured by four items, and another four items were used to measure work interference with family. Responses included “not at all” (coded as 0), “to some extent” (coded as 1), “a great deal” (coded as 2), and “not applicable or don’t have a family” (coded as 0). These scales were adapted from the National Study of Midlife Development in the United States and have been shown to be reliable and valid (Netemeyer, Boles, & McMurrian, 1996). Questionnaires are shown in the online Supplementary Material.

Answers were scored from 0 to 8, so that a higher score reflected greater conflict (treated as a continuous variable in the model). For all items inferring about work–family conflict, participants could indicate “not applicable or don’t have a family,” for example, when participants did not have a family or were (temporarily) out of work. Participants who reported “not applicable” for all items of FIW and WIF at a particular phase received score 0. To distinguish “not applicable” from those who perceived the lowest level of conflict, we additionally included a binary variable in the model, which was coded as 1 for the “not applicable” situations and 0 otherwise.

#### Confounders

Demographic characteristics, including highest education, employment grade, whether still working in the civil service, and spouse’s work status were adjusted for as potential confounders. Highest educational qualification was measured in three categories, “low” (General Certificate of Education O-level or lower; lower secondary education or lower), “middle” (General Certificate of Education A-level or equivalent; upper secondary education), and “high” (Degree level; tertiary education). Information on highest educational qualification was only collected in phase 5; in case it was missing, we used information from phase 1 on the age of finishing full-time education. Employment grade levels at the civil service are: “Administrative” (highest), “Professional/Executive” (middle), or “Clerical/Support” (lowest), which were measured at phases 3, 5, and 7. For participants who were working outside the civil service, their last employment grade before leaving civil service was used. We adjusted for whether someone was still working in the civil service, because participants moving to employment outside the civil service might have different pension regulations. We included time-varying spouse’s work status (no partner, partner in work, partner not in work). It was measured at phases 1, 5, and 7, and we used phase 1 to replace phase 3.

#### Potential mediators

Increased work–family conflict may lead to poor health of employees, which is an important determinate of work exit (Karpansalo, Manninen, Kauhanen, Lakka, & Salonen, 2004). Thus, work–family conflict may have indirect influences through health on work exit. Depressive symptoms and number of chronic conditions at each phase 3, 5, and 7 were included as potential mediators. Symptoms of depression were measured by the General Health Questionnaire (GHQ), and cutoff points of 4 of 12 were used to identify depression case (Singh-Manoux et al., 2010). Number of chronic conditions (ranging from 0 to 4) was measured using validated information on the onset of diabetes, coronary heart disease, all malignant cancers, and all stroke. Details of how chronic conditions were validated can be found elsewhere (Fleischmann et al., 2017).

#### Sources of work–family conflict

Stressful working conditions and family characteristics could be the sources of work–family conflict and were treated as time-varying at phase 3, 5, and 7. Psychosocial working conditions were derived from the Karasek’s (1979) questionnaires for the job strain model, including job demand, decision latitude, and social support at work. Job demands were operationalized by four items such as “Do you have to work very fast?” Decision latitude was measured by 15 items (nine for decision authority and six for skill discretion), such as “Do you have a choice in deciding how to do your work?” Social support at work consisted of six items combining aspects of support from colleagues. Items were added up, and higher values indicate higher levels of job demands, decision latitude, and social support. These continuous scores were divided into tertiles (low, middle, and high).

Family factors included were the number of dependent children (0, 1, 2, or more) under age 18 in the household (only measured at phase 5), caring for aged/disabled relative (measured at phases 3, 4, and 7, and we used phase 4 to replace phase 5), and control at home (phases 3, 5, and 7). Control at home was measured by answering “At home, I feel have control over what happens in most situations” from “strongly agree” to “strongly disagree” (6-point scale). “Strongly disagree” and “moderately disagree” were combined due to few observations in these categories.

### Statistical Methods

Cause-specific Cox models were applied to investigate the time to work exit through different routes (*stcox* in Stata). We used cause-specific hazard with “standard” survival analysis and treated the other event as censored (Lau, Cole, & Gange, 2009). For example, when retirement is the event of interest, people who are unemployed were only at risk of retirement before they became unemployed, and their follow-up was censored at unemployment. In all models, age was used as the timescale. We tested the proportional hazards assumption by introducing an interaction between WIF/ FIW and time. Interactions were not statistically significant; thus, the proportional hazards assumption is met.

Work–family conflict may change over time; therefore, we treated work–family conflict as a time-varying variable (phases 3, 5, and 7). The hazard of work exit between phases 3 and 5 was regressed on work–family conflict at phase 3, whereas the hazard between phases 5 and 7 was regressed on conflict at phase 5, and the hazard between phases 7 and 11 was regressed on conflict at phase 7.

Missing data of covariates and work–family conflict (i.e. have one or two time-points measures, but not all three) were imputed in Stata, using multivariate imputation by chained equations, and 30 data sets were imputed. We included all variables from the analyses (i.e. independent variables, outcome variables, and covariates) in the imputation model.

For each route of exit, we first show the raw hazard ratio (HR) from Cox regressions (Model 1). Model 2 adjusted for confounders (including highest education, employment grade, still in the civil service, and employment of spouse/no spouse). In Model 3, we included potential mediators (GHQ depression and number of chronic conditions) to assess whether work–family conflict could influence work exit indirectly through these factors. We additionally adjusted for psychosocial working conditions in Model 4, and for family factors in Model 5 (without adjusting for working conditions). In Model 6, both working conditions and family factors were included as sources of work–family conflict.

Finally, we show how work conditions and family factors are related to work exit. We do this to better understand which factor is a particularly important source of work–family conflict that may lead to work exit.

## Results

Both observed and imputed sample characteristics as well as missing percentage for each variable at baseline (phase 3) are shown in Table 1. Missing percentage ranged from 0% to more than 20%, with the highest levels of missing data for spouse’s work status (20.6% for men, 16.6% for women). Imputed data showed very similar patterns as observed data, and thus we only reported the imputed characteristics here. More than 70% of men and women were still working in the civil service rather than working outside. More men than women had “high” levels of educational qualification (36.3% vs 24.0% “Degree level or higher”) and the highest employment grade (48.4% vs 17.7% “Administrative”). More men (23.1% vs 6.1%) had a nonworking spouse, but more women did not have a spouse (40.8% vs 19.9%). Among men and women, on average, one in seven had a raised GHQ depression score, and less than 7% had chronic conditions. Compared to women, men were more likely to report high job demands (35.1% vs 26.6%) and high decision latitude (50.4% vs 28.5%), but men and women reported similar levels of social support at work. More women had caring responsibilities (13.6%) than men (9.6%), but more men (29%) than women (13%) had dependent children in the household. Women reported higher control at home than men. On average, men scored 2.92 (*SD* = 1.81) on the WIF scale and women scored 2.25 (*SD* = 1.71). The mean value on the FIW scale was 1.64 (*SD* = 1.57) for men and 1.68 (*SD* = 1.66) for women.

**Table 1.** Descriptive Characteristics of Study Sample at Phase 3

	Men (*N* = 5,157)			Women (*N* = 2,027)		
	Observed *n* (% missing)	Observed %	Imputed %	Observed *n* (% missing)	Observed %	Imputed %
Highest education	4,937 (4.3)			1,881 (7.2)		
Low		37.0	37.0		56.4	56.6
Middle		26.7	26.7		19.3	19.4
High		36.3	36.3		24.3	24.0
Employment grade	5,157 (0)			2,027 (0)		
Clerical/support		6.3	6.3		37.3	37.3
Professional/executive		44.9	44.9		45.0	45.0
Administrative		48.8	48.8		17.7	17.7
Working in civil service (rather than working outside)	4,951 (4.0)	70.9	70.7	1,925 (5.0)	72.3	72.0
Spouse’s working status	4,094 (20.6)			1,691 (16.6)		
Working spouse		56.1	57.0		53.7	53.1
Nonworking spouse		22.4	23.1		4.0	6.1
No spouse		21.5	19.9		42.3	40.8
GHQ depression	4,989 (3.3)	12.6	12.6	1,973 (2.7)	14.2	14.3
Number chronic conditions	5,157 (0)			2,027 (0)		
0		93.6	93.6		93.2	93.2
1		6.2	6.2		6.6	6.6
2+		0.2	0.2		0.2	0.2
Job demand	4,919 (4.6)			1,949 (3.9)		
Low		18.4	18.8		28.9	29.0
Middle		46.2	46.1		44.4	44.4
High		35.4	35.1		26.7	26.6
Job decision latitude	4,917 (4.7)			1,948 (3.9)		
Low		19.2	19.3		39.5	39.4
Middle		30.2	30.3		32.1	32.1
High		50.6	50.4		28.4	28.5
Social support at work	4,868 (5.6)			1,936 (4.5)		
Low		34.1	34.1		34.3	34.3
Middle		33.9	33.9		31.6	31.6
High		32.0	32.0		34.1	34.1
Number dependent children						
0	4,355 (15.6)	71.5	70.5	1,636 (19.3)	87.0	87.1
1		12.5	12.9		7.3	6.7
2		11.4	11.3		4.2	4.5
3+		4.6	5.3		1.5	1.7
Caring responsibility	4,991 (3.2)	9.4	9.4	1,973 (2.7)	13.5	13.6
Have control at home	4,989 (3.3)			1,973 (2.7)		
Strongly agree (highest)		31.6	31.6		45.0	45.0
Moderately agree		47.5	47.5		41.2	41.2
Slightly agree		8.6	8.6		5.0	5.0
Slightly disagree		6.0	6.0		3.7	3.7
Strongly/moderately disagree		6.3	6.3		5.1	5.1
WIF (range 0–8)	4,854 (5.9)	2.93 (1.81)^a^	2.92 (1.81)^b^	1,871 (7.7)	2.22 (1.71)^a^	2.25 (1.71)^b^
FIW (range 0–8)		1.64 (1.57)^a^	1.64 (1.57)^b^		1.67 (1.66)^a^	1.68 (1.66)^b^

*Note*. GHQ = General Health Questionnaire; FIW = family interference with work; WIF = work interference with family; *SD* = standard deviation.

^a^Values are observed mean (*SD*).

^b^Values are imputed mean (*SD*).

Table 2 shows participants’ main route and age of work exit. A total of 71.5% of men and 76.5% of women left work during follow-up. Among those who have exited the labor market, retirement was the most frequent transition out of work (83.3% men and 79.8% women). More women than men left work through the “health-related” (9.6% vs 7.6%) and the “homemaker/other” route (7.2% vs 4.6%). Slightly more men (4.5%) left work due to unemployment than women (3.4%). Average age at work exit (among those who have exited) was 60.33 years (*SD* = 4.93) for men and 59.63 (*SD* = 4.65) for women. Those leaving for retirement were on average the oldest (60.87 and 60.41 for men and women, respectively). Female homemakers/other were youngest when leaving work, on average 56.25 years.

**Table 2.** Route and Age of Work exit for Men and Women.

	Men (*N* = 5,157)		Women (*N* = 2,027)	
	%	Mean age at censored/exit (*SD*)	%	Mean age at censored/exit (*SD*)
Censored	28.5	62.00 (7.28)	23.5	61.27 (6.70)
Exit work	71.5	60.33 (4.93)	76.5	59.63 (4.65)
Route of exit^a^				
Retirement	83.3	60.87 (4.69)	79.8	60.41 (4.01)
Health-related	7.6	57.51 (5.36)	9.6	56.81 (5.66)
Unemployment	4.5	57.11 (4.99)	3.4	56.66 (6.22)
Homemaker/other	4.6	58.51 (5.13)	7.2	56.25 (5.44)

^a^Among those who have exited work in the follow-up. *n* = 3,686 for men, *n* = 1,551 for women. *SD* = standard deviation

We found that the association between WIF and retirement/homemaking exits, and between FIW and homemaking differed significantly by gender (results are not shown). We thus show the association between WIF/FIW and work exit for men and women separately in Table 3. For men, WIF was not associated with all routes combined exit in any of the models, but FIW was associated with decreased risk of all routes combined exit after adjusting for confounders. For women, neither WIF nor FIW was associated with all routes combined work exit after adjusting for confounders.

**Table 3.** Cause-Specific Cox Models for the Relationship Between Work–Family Conflict (WIF and FIW) and Exit From Work

	All routes combined ^a^				Retirement				Homemaker/other route			
	Men (*n* = 3,686/5,157)		Women (*n* = 1,551/2,027)		Men (*n* = 3,072/5,157)		Women (*n* = 1,237/2,027)		Men (*n* = 170/5,157)		Women (*n* = 112/2,027)	
	HR	95% CI	HR	95% CI	HR	95% CI	HR	95% CI	HR	95% CI	HR	95% CI
WIF												
Model 1	0.99	0.96 to 1.02	1.10***	1.04 to 1.15	0.99	0.96 to 1.02	1.09**	1.03 to 1.14	0.96	0.84 to 1.10	1.30**	1.10 to 1.54
Model 2	0.98	0.95 to 1.01	1.04	0.99 to 1.10	0.98	0.95 to 1.01	1.04	0.98 to 1.10	1.00	0.86 to 1.16	1.19	0.98 to 1.44
Model 3	0.98	0.95 to 1.01	1.04	0.99 to 1.10	0.98	0.95 to 1.01	1.04	0.98 to 1.10	0.98	0.84 to 1.14	1.22*	1.00 to 1.48
Model 4	0.98	0.95 to 1.02	1.06	0.99 to 1.12	0.97	0.94 to 1.01	1.05	0.99 to 1.12	0.98	0.83 to 1.15	1.17	0.95 to 1.44
Model 5	0.97	0.94 to 1.00	1.02	0.96 to 1.08	0.97	0.94 to 1.00	1.02	0.96 to 1.09	0.95	0.82 to 1.11	1.14	0.94 to 1.40
Model 6	0.98	0.94 to 1.01	1.04	0.97 to 1.10	0.97	0.94 to 1.00	1.04	0.97 to 1.11	0.95	0.80 to 1.12	1.10	0.88 to 1.37
FIW												
Model 1	0.97	0.94 to 1.00	1.03	0.97 to 1.08	0.97	0.93 to 1.00	1.00	0.95 to 1.06	0.91	0.77 to 1.08	1.26**	1.07 to 1.49
Model 2	0.95**	0.92 to 0.99	1.01	0.96 to 1.07	0.95*	0.92 to 0.99	0.99	0.93 to 1.05	0.92	0.78 to 1.10	1.23*	1.03 to 1.47
Model 3	0.95**	0.92 to 0.99	1.01	0.96 to 1.07	0.95**	0.92 to 0.99	0.99	0.93 to 1.05	0.89	0.75 to 1.06	1.27*	1.06 to 1.52
Model 4	0.95**	0.92 to 0.98	1.01	0.96 to 1.07	0.94**	0.91 to 0.98	0.99	0.93 to 1.05	0.89	0.74 to 1.06	1.23*	1.03 to 1.48
Model 5	0.92***	0.89 to 0.96	0.98	0.92 to 1.04	0.93***	0.89 to 0.96	0.97	0.90 to 1.03	0.82*	0.68 to 0.99	1.12	0.91 to 1.37
Model 6	0.92***	0.89 to 0.96	0.98	0.92 to 1.04	0.92***	0.89 to 0.96	0.97	0.91 to 1.04	0.82*	0.68 to 0.99	1.09	0.88 to 1.34

*Note*. Model 1: Cause-specific Cox models using age as the timescale. Binary variable indicating N/A in work–family conflict was included. Model 2: model 1 + confounders (including highest education, employment grade, whether still in the civil service, employment of spouse/no spouse). Model 3: model 2 + potential mediators (General Health Questionnaire depression and number of chronic conditions). Model 4: model 3 + psychosocial working conditions (including job demands, job decision latitude, and support at work). Model 5: model 3 + family related factors (including number of dependent children in the household, caring responsibility, and control at home). Model 6: model 3 + psychosocial working conditions and family related factors. FIW = family interference with work; WIF = work interference with family; HR = hazard ratio; CI = confidence interval.

^a^Routes exit from work are retirement, homemaker/other exit, health-related exit, and unemployment. Results of health-related exit and unemployment are not significant and are shown in Supplementary Material.

*p < .05. **p < .01. ***p < .001.

When looking at particular routes of work exit, WIF was not significantly associated with any type of work exit for men in any model. In terms of FIW, the risk of retirement among men decreased by 5% (HR = 0.95, 95% confidence interval [CI] = 0.92 to 0.99) with every one unit increase in FIW scale, after adjusting for confounders (Model 2). Adding health did not change the association (Model 3). Including psychosocial working conditions and family factors or both made this association among men even stronger, rather than attenuating it (Models 4–6). Men with higher FIW were also less likely to exit to be a homemaker/other, after taking account of family factors (Model 5).

In the raw model, women with higher WIF scores were more likely to exit work through retirement (HR = 1.09, 95% CI = 1.03 to 1.14) and homemaker/other route (HR = 1.30, 95% CI = 1.10 to 1.54), but associations were no longer significant after adjusting for confounders (Model 2). Women with higher FIW scores were more likely to exit work through the homemaker/other route (Model 1 and 2). The increased risk of exit through homemaker/other route was not attenuated by adding health (Model 3) or working conditions (Model 4), but adjusting for family factors made women’s increased risk of exit was no longer significant (Model 5). Neither FIW nor WIF was significantly associated with health-related work exit or unemployment (results are shown in Supplementary Table 1).

We did a sensitivity analysis by using four quadrants of job strain (high demand and high decision latitude, high demand and low decision latitude, low demand and high decision latitude, and low demand and low decision latitude) to combine job control and decision latitude, and it did not change our results in Table 3 (analysis results not shown).

Table 4 shows the association of psychosocial working conditions and family factors with work exit through retirement and homemaker/other route (without adjusting for work–family conflict). Men and women with one or two dependent children, rather than none, were more likely to retire. Men with a high, rather than low, job decision latitude were less likely to retire, but psychosocial working conditions were not related to women’s retirement. Men and women with lower home control (compared to the highest), men with three or more dependent children, and women with caring responsibilities were more likely to make the homemaker/other exit.

**Table 4.** Cause-Specific Cox Models for the Relationship Between Sources of Work–Family Conflict and Exit From Work^a^

	Men				Women			
	Retirement		Homemaker/other		Retirement		Homemaker/other	
	HR	95% CI	HR	95% CI	HR	95% CI	HR	95% CI
Job demand								
Low	Ref		Ref		Ref		Ref	
Middle	1.07	0.93 to 1.23	1.02	0.56 to 1.87	0.93	0.73 to 1.19	2.80	0.97 to 8.07
High	1.02	0.86 to 1.20	1.09	0.53 to 2.28	0.82	0.62 to 1.09	2.49	0.77 to 8.10
Job decision latitude								
Low	Ref		Ref		Ref		Ref	
Middle	1.02	0.86 to 1.21	0.81	0.39 to 1.67	1.09	0.85 to 1.39	1.21	0.48 to 3.02
High	0.76**	0.64 to 0.90	0.74	0.35 to 1.54	0.93	0.70 to 1.24	0.99	0.39 to 2.47
Social support at work								
Low	Ref		Ref		Ref		Ref	
Middle	0.98	0.85 to 1.13	1.62	0.88 to 3.00	0.97	0.77 to 1.24	0.87	0.39 to 1.97
High	1.02	0.89 to 1.17	1.25	0.64 to 2.46	0.87	0.67 to 1.12	0.86	0.38 to 1.94
Dependent children								
0	Ref		Ref		Ref		Ref	
1	1.35***	1.16 to 1.55	1.20	0.60 to 2.42	1.56***	1.16 to 2.10	1.20	0.46 to 3.14
2	1.37***	1.18 to 1.61	1.31	0.65 to 2.65	2.10***	1.44 to 3.08	1.14	0.36 to 3.61
3+	1.02	0.79 to 1.31	2.25*	1.00 to 5.06	1.33	0.65 to 2.70	2.89	0.66 to 12.64
Caring responsibility	1.07	0.92 to 1.25	1.32	0.69 to 2.54	0.93	0.72 to 1.20	3.25***	1.78 to 5.94
Have control at home								
Strongly agree (highest)	Ref		Ref		Ref		Ref	
Moderately agree	1.06	0.94 to 1.19	1.06	0.58 to 1.95	1.14	0.79 to 1.66	1.13	0.55 to 2.30
Slightly agree	1.07	0.88 to 1.29	1.16	0.47 to 2.84	0.87	0.54 to 1.39	1.98	0.69 to 5.70
Slightly disagree	1.01	0.80 to 1.27	2.92**	1.36 to 6.26	1.12	0.77 to 1.62	3.48***	1.33 to 9.12
Strongly/moderately disagree (lowest)	1.05	0.93 to 1.32	1.19	0.43 to 3.27	1.03	0.84 to to 1.27	0.82	0.19 to 3.64

*Note*. HR = hazard ratio; CI = confidence interval.

^a^Other covariates included are highest education, employment grade, still in the civil service, employment of spouse/no spouse, General Health Questionnaire depression, and number of chronic conditions.

*p < .05. **p < .01. ***p < .001.

## Discussion

In this study, we investigated how work–family conflict was related to routes of work exit in the later career stage. Our results suggest that work–family conflict may influence men’s and women’s work exit differently. Men were less likely to exit work (through retirement and homemaker/other route) when they felt family interfered with work (FIW). Women, on the other hand, were more likely to become a homemaker/other when family interfered with work. Because the “homemaker/other” route only accounts for a small proportion of work exit, FIW was no longer related to women’s work exit when combining all routes of exit. WIF played a less important role in work exit, as it only increased the risk of exit (through retirement and homemaker/other route) for women before adjusting for confounders, and it was not associated men’s labor force participation in any model.

Our study partly supports Hypothesis 1 that higher work–family conflict is an indicator for work exit by showing that FIW was associated with increased risk of exit through the “homework/other” route among women. We also found that family factors including caring responsibility and lower control at home made this association no longer significant among women. This could indicate that high family demand was the source of work–family conflict for women (Hypothesis 4).

On the other hand, we found a negative association between FIW and work exit for men. Adjusting for psychosocial working conditions and family factors did not explain the increased risk of remaining in work among those with high FIW, and even made the association stronger. This is contrary to our first hypothesis. Two explanations for this unexpected finding seem reasonable. First, FIW may (partly) capture internal career orientation. In our study, men who reported high FIW could differ with regard to other (unmeasured) family characteristics, such as marital satisfaction. If marital discord is causing stress, staying at home does not solve the work–family conflict, but instead, work might be a “haven” from the stressful family sphere (Raymo & Sweeney, 2006). This explanation would be partly consistent with “compensation” theory, which points out that individuals with unsatisfying family lives might turn to other spheres to achieve satisfaction (Edwards & Rothbard, 2000). Second, the more important a role is to an individual, the more time and energy that person will spend on it, and thus expend less effort in other roles (Greenhaus & Beutell, 1985). Men with higher FIW could be those with a very salient work role, causing them to be more likely to stay in work rather than taking domestic labor when there is a conflict between work and family. This is possibly the reason why adjusting for working conditions and family factors made the association stronger. On a similar note, higher WIF might be indicative of a more salient family role, but we did not find an association between WIF and work exit among men. This is probably because the “male breadwinner” role is dominant among this population (McMunn et al., 2015), and men with higher WIF may have to continue to work for financial reasons.

Our findings are in line with the second hypothesis that women are more likely to exit work than men when there is a work and family conflict. It is possible that where there is a work–family conflict for both partners, there may be a decision for one to leave work, and it is more likely that women will be the ones to compromise and to leave the work. As women have fewer financial resources (including pension wealth) and on average contribute fewer earnings to the household than men, they may therefore have less “bargaining power” on work decisions than their male partner (Jia, 2005; van der Horst, Lain, Vickerstaff, Clark, & Baumberg Geiger, 2017). We were not able to measure this “bargaining power” on work exit decisions, but our results (Table 4) show that reporting lower control at home was associated with higher risk of exit through the “homemaker/other” route for both men and women, and the association was stronger for women.

Contrary to our third hypothesis, GHQ depression and number of chronic conditions did not mediate the association between work–family conflict and work–exit. It is possible that health conditions are more important for those who exit through the health-related route, but our study did not find any significant association between work–family conflict and health-related work exit.

Most previous studies captured work exit intentions rather than actual work exit behavior and have linked higher work–family conflict to increased work exit intentions (Forma, 2009; Garcia et al., 2014; Raymo & Sweeney, 2006). However, actual work exit behavior might be different from intentions, especially when investigating more permanent or final work exit in later life. Relatedly, Greenhaus and Parasuraman (2001) reported that WIF was associated with increased withdrawal intention, but not with withdrawal behavior. Kubicek and colleagues (2010) studied the probability of retiring earlier than age 62 in the United States and found that higher FIW decreased the probability of retiring early, but higher WIF increased the risk. Our study focuses on the risk of retirement across the late career stage rather than dichotomizing the outcome as retirement before the state/occupational pension age. Our findings of the association between FIW and the risk of retirement for men are partly consistent with their study.

Some situations in life that cause conflicts between work and family are only temporary. For example, caring for an older relative may be incompatible with work and increases work–family conflict. However, once the caring responsibility ends, the conflict will be reduced or disappear. Previous studies, relying on single time-point measures, have not taken this into account. In contrast, we incorporated the changing nature of work–family conflict by using three repeated measures, spanning more than 10 years, to predict the risk of work exit. Our study has several other strengths, including a large study sample, detailed family characteristics, working conditions, and employment data. We distinguished different routes of work exit, to better understand the mechanisms how work–family conflict links to work exit. However, we need to consider several limitations of this study. The Whitehall II study uses a sample of civil servants in London and is not representative of the general population. We did not include people who were already out of the labor market at the onset of the study, and some of those may have exited work due to work–family conflicts. Income and wealth were not available, but we did include employment grade, which is an important indicator of socioeconomic position.

In conclusion, this study is the first to consider both WIF and FIW effects on work exit in the United Kingdom. FIW was associated with higher risk of exit through the homemaker/other route among women but decreased the risk of exiting work among men. WIF played a less important role in determining work exit. Incompatibility of family and work demands may reinforce gender inequality because women who are experiencing intense conflict and cultural pressure may have to fully devote themselves to domestic work. These women may have accumulated fewer years of contributions than their counterparts who continued working, with direct implications for their pension wealth. Adjustments in the workplace, such as flexible working hours and higher social support, could reduce work–family conflict and help these women to remain in work longer (Kelly et al., 2014). Work–family conflict was thought to be an important issue for younger people with children, but our study underscores the importance of work–family conflict for older people’s labor market participation. Considering that women have a much younger retirement age than men, and that the pressures on social benefits systems are increasing in the context of demographic aging, it is important to reduce work–family conflict for women across working life.

## Supplementary Material

gby146_suppl_Supplementary_Material
