# Supplementary material for: Work-Family Conflict and Work Exit in Later Career Stage
Source: J Gerontol B Psychol Sci Soc Sci. 2018 Nov 28;75(3):716–27. doi: 10.1093/geronb/gby146 (PMC7768697; doi:10.1093/geronb/gby146)
Supplement: gby146_suppl_Supplementary_Material [file gby146_suppl_supplementary_material.docx]

**Supplementary Material**

**Questions used to measure work-family conflict**

To what extent do your family life and family responsibilities interfere with your job?

Would you say:

1. Family matters reduce the time you can devote to your job
2. Family worries or problems distract you from your work
3. Family activities stop you getting the amount of sleep you need to do your job well
4. Family obligations reduce the time you need to relax or be by yourself

To what extent do your job responsibilities interfere with your family life?

Would you say:

1. Your job reduces the amount of time you can spend with the family
2. Problems at work make you irritable at home
3. Your job involves a lot of travel away from home
4. Your job takes so much energy you don't feel up to doing things that need attention at home

Supplementary Table 1. Cause-specific Cox models for the relationship between work-family conflict (WIF& FIW) and health-related exit work and unemployment.

|  | **Health-related route** | | | |  | **Unemployment** | | | |
| --- | --- | --- | --- | --- | --- | --- | --- | --- | --- |
|  | **Men (n=279/5157)** | | **Women(n=149/2027)** | |  | **Men (n=165/5157)** | | **Women(n=53/2027)** | |
|  | **HR** | **95%CI** | **HR** | **95%CI** |  | **HR** | **95%CI** | **HR** | **95%CI** |
| **WIF** |  |  |  |  |  |  |  |  |  |
| Model 1 | 0.89 | 0.78, 1.02 | 0.90 | 0.72, 1.15 |  | 1.10 | 0.94, 1.30 | 1.29 | 0.93, 1.79 |
| Model 2 | 0.95 | 0.82, 1.10 | 0.87 | 0.67, 1.14 |  | 1.18 | 0.99, 1.41 | 1.25 | 0.87, 1.79 |
| Model 3 | 0.94 | 0.81, 1.09 | 0.84 | 0.64, 1.12 |  | 1.18 | 0.99, 1.41 | 1.23 | 0.85, 1.77 |
| Model 4 | 0.98 | 0.84, 1.15 | 0.87 | 0.64, 1.18 |  | 1.18 | 0.98, 1.42 | 1.21 | 0.81, 1.80 |
| Model 5 | 0.93 | 0.80, 1.09 | 0.81 | 0.60, 1.09 |  | 1.13 | 0.95, 1.36 | 1.20 | 0.82, 1.76 |
| Model 6 | 0.98 | 0.83, 1.15 | 0.85 | 0.62, 1.16 |  | 1.13 | 0.93, 1.37 | 1.18 | 0.78, 1.78 |
| **FIW** |  |  |  |  |  |  |  |  |  |
| Model 1 | 0.97 | 0.83, 1.13 | 0.93 | 0.71, 1.22 |  | 1.12 | 0.94, 1.34 | 1.27 | 0.92, 1.75 |
| Model 2 | 0.97 | 0.84, 1.13 | 0.94 | 0.71, 1.24 |  | 1.13 | 0.96, 1.34 | 1.27 | 0.91, 1.76 |
| Model 3 | 0.94 | 0.81, 1.11 | 0.92 | 0.69, 1.23 |  | 1.13 | 0.94, 1.34 | 1.24 | 0.88, 1.73 |
| Model 4 | 0.96 | 0.82, 1.13 | 0.93 | 0.70, 1.24 |  | 1.10 | 0.92, 1.32 | 1.22 | 0.86, 1.71 |
| Model 5 | 0.93 | 0.79, 1.10 | 0.92 | 0.68, 1.24 |  | 1.02 | 0.85, 1.23 | 1.21 | 0.83, 1.76 |
| Model 6 | 0.95 | 0.80, 1.12 | 0.94 | 0.69, 1.27 |  | 1.02 | 0.84, 1.23 | 1.19 | 0.82, 1.74 |

Model 1: Cause-specific cox models using age as the timescale. Binary variable indicating N/A in work-family conflict was included.

Model 2: model 1 + confounders (including highest education, employment grade, whether still in the civil service, employment of spouse/no spouse)

Model 3: model 2 + potential mediators (GHQ depression and number of chronic conditions)

Model 4: model 3 + psychosocial working conditions (including job demands, job decision latitude, and support at work)

Model 5: model 3 + family related factors (including number of dependent children in the household, caring responsibility, and control at home)

Model 6: model 3 + psychosocial working conditions and family related factors.
